# Dosimetry and Feasibility Studies of Volumetric Modulated Arc Therapy With Deep Inspiration Breath-Hold Using Optical Surface Management System for Left-Sided Breast Cancer Patients

**DOI:** 10.3389/fonc.2020.01711

**Published:** 2020-09-03

**Authors:** Wei Zhang, Ruisheng Li, Dong You, Yi Su, Wei Dong, Zhao Ma

**Affiliations:** ^1^Department of Radiation Oncology, Yantai Yuhuangding Hospital, Qingdao University, Yantai, China; ^2^Department of Medical Imaging, Yantai Yuhuangding Hospital, Yantai, China

**Keywords:** DIBH, OSMS, breast cancer, radiotherapy, VMAT

## Abstract

**Background:**

During radiotherapy (RT) procedure of breast cancer, portions of the heart and lung will receive some radiation dose, which may result in acute and late toxicities. In the current study, we report the experience of our single institution with organs at risk (OARs)–sparing RT with deep inspiration breath hold (DIBH) using an Optical Surface Management System (OSMS) and compare the dosimetric parameters with that of free breathing (FB).

**Patients and Methods:**

Forty-eight cases diagnosed as early stage left-sided breast cancer scheduled for postoperative RT were enrolled. The OSMS was used to monitor the breathing magnitude and track the real-time respiratory status, which can control a stable lung and heart volume during RT delivery under DIBH. We did the dosimetric analysis of the heart, left anterior descending (LAD) coronary artery, lungs, and contralateral breast under FB and DIBH plans.

**Results:**

Compared with FB–volumetric-modulated arc therapy (FB-VMAT), DIBH-VMAT resulted in significantly changed volumes to the heart and lungs receiving irradiation dose. The average mean heart dose and average D2%, V_5_, and V_10_ showed significant differences between the DIBH and FB techniques. For the LAD coronary artery, we found significantly reduced average mean dose, D2%, and V_10_ with DIBH. Similar results were also found in the lungs and contralateral breast. The use of flattening-filter–free decreased treatment time compared with the flat beam mode in our VMAT (*p* < 0.05). For the 48 patients, there were no significant differences in the lateral, longitudinal, and vertical directions between OSMS and cone beam CT.

**Conclusions:**

DIBH-VMAT with OSMS is very feasible in daily practice with excellent patient compliance in our single-center experience. Note that OSMS is an effective tool that may allow easier-to-achieve precise positioning and better and shorter position-verify time. Meanwhile, compared with FB, DIBH was characterized by lower doses to OARs, which may reduce the probability of cardiac and pulmonary complications in the future.

## Introduction

As the most common cancer in women globally, breast cancer has a high 5-year overall survival rate of 90% attributed to advances in prevention, early diagnosis, and treatment regimens, including radiotherapy (RT) (1). However, adjuvant RT can result in some notable acute (e.g., skin and esophageal toxicity) and late (e.g., cardiovascular toxicity, lung fibrosis, and secondary cancers) adverse events (2–6). In a famous study, Darby et al. (5) reported that during breast cancer RT, the heart was exposed to ionizing radiation, resulting in an increase in ischemic heart disease. Heart disease starts several years after RT and will last for no less than 20 years. For cases with already existed cardiac risk factors undergoing RT, the increase is significantly higher than in other patients. Among these risk factors, cardiovascular disease (CVD) is a major complication and a serious death risk for breast cancer survivors (7–9). A recent systematic review from Gernaat et al. (10) provided strong evidence about elevated CVD-related mortality risk in breast cancer. In summary, there is an urgent need to decrease irradiation dose of the heart in patients receiving RT, especially for those with left-sided breast cancer.

Patient holds it for a prolonged time after deep inspiration under deep inspiration breath hold (DIBH) technique for the administration of simulation and RT. During the DIBH procedure, expansion of the lungs and movement of the diaphragm facilitate the heart a longer distance from the chest wall and then away from photon beam transmission. During the simulation procedure and treatment administration, the patient holds breath when radiation is administered. Several published studies on DIBH have shown that (11–15) it is a technique with good feasibility and reproducibility that helps in reducing the irradiated heart volume, mean heart dose, and mean left anterior descending (LAD) coronary artery dose.

Currently, there are three methods in clinical application to maintain DIBH, including active breathing coordinator, real-time position management, and voluntary breath-hold. The Optical Surface Management System (OSMS) constitutes more advanced tools (e.g., AlignRT, Vision RT Ltd., London, United Kingdom; Sentinel, C-RAD, Uppsala, Sweden) in vDIBH (16). As we reported previously (17), in the OSMS, three-dimensional (3D) surface reconstruction can be visualized by the stereovision to align to the isocenter, providing a real-time position monitoring.

Breast intensity-modulated radiation therapy (IMRT) has gained interest that resulted from its feasibility, dosimetric superiority, decreased acute side effects, few late complications, and reduced skin toxicity (18–22). Volumetric-modulated arc therapy (VMAT) is an extension of IMRT that can achieve a decreased treatment time and increase normal tissue dose sparing in patients diagnosed with prostate, head and neck, and lung cancers (23). Several other studies have also shown that the VMAT technique can shorten treatment delivery times relative to the IMRT technique and provide a similar planning target volume (PTV) coverage and organs at risk (OARs) sparing in prostate, cervix, and head and neck cancer cases (24–26), while for early stage left-sided breast cancer patients undergoing postoperative RT with DIBH technique, the utilization of VMAT technique can result not only in lower delivered monitor units (MU), but also shorter treatment time (27).

To our knowledge, studies comparing DIBH-VMAT with free breathing (FB)–VMAT using OSMS techniques are rare. Therefore, we aimed to analyze the dosimetric comparison of postoperative RT using the above techniques in patients with left-sided breast-conserving surgery. The hypothesis was that DIBH-VMAT would be superior to or equivalent to FB-VMAT in terms of sparing OARs, especially the heart and LAD coronary artery. In addition, based on the higher accuracy, shorter position-verify time, and lower radiation dose with the OSMS, this study assessed the benefit of DIBH using OSMS-based VMAT in early stage patients with postoperative adjuvant RT after left breast-conserving surgery.

## Materials and Methods

### Patient Selection

In the current study, only patients with good performance status (Eastern Cooperative Oncology Group performance status 0–1) and an excellent understanding and exhibition of the DIBH process and younger than 70 years were selected. Between January 2018 and June 2019, 48 consecutive patients with adjuvant RT after left-sided breast-conserving surgery were enrolled. All patients’ understanding and sign of written informed consent should be checked before treatment.

### Computed Tomography Simulation

**FIGURE 1:**
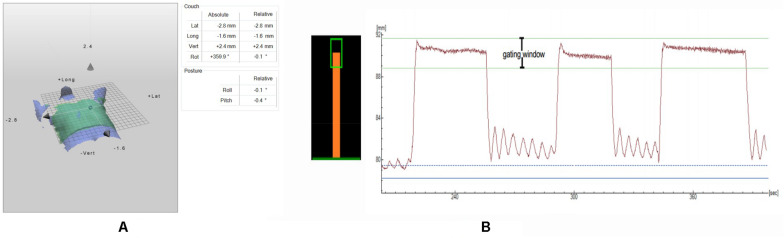
**(A)** The calculation of the setup errors by the OSMS registration; **(B)** Left: Visual feedback of the breathing position for the patient: gating window (green box) and breathing position (orange bar); Right: A DIBH breathing curve from CT scanning, with gaps of free breathing between each DIBH.

Before the start, we interpreted the flowchart concisely to the enrolled patients and trained them with DIBH in detail to ensure perfect implementation during the application of the OSMS. During application of the OSMS, we used Smart Glasses (H756A, SEIKO EPSON Corp., Philippines), by which the patient can see the feedback of its own breathing curve. The respiratory observation site is located near the diaphragm. The amplitude of movement in this position during breathing is obvious and easy to monitor. Additionally, the software calculates an average (breathing amplitude) of the patient’s free, steady breathing cycles, which serve as the patient’s baseline “*a*.” Then, the patient was asked to breath-hold first, the breathing range was observed during breath-holding, and the process was repeated three times to obtain an average breath-hold value of “*b*,” and *b* minus “*a*” yielded the value “*c*.” The range from *c* - 1.5 mm to *c* + 1.5 mm was set as the gating window (as shown in Figure 1B). After the patient exhibited a good DIBH status, two consecutive computed tomography (CT) simulation scans in 5-mm slices (FB and DIBH scans; Discovery RT590; GE Healthcare, Waukesha, WI, United States) were acquired in the same position. All scans were taken in the supine position with two arms over heads on the breast board (R610-DCF1, Klarity, China).

### Contouring

After the CT scan, the CTVs and OAR were delineated on each FB and DIBH scan based on “the Radiation Therapy Oncology Group guidelines (28). The PTV is a 5-mm extension of the CTV but is limited to 3 mm underneath the skin. All relevant OARs were delineated, including the heart, the LAD coronary artery, the ipsilateral lung, the contralateral lung, and the contralateral breast. When contouring the heart, all the heart muscles including the complete pericardia bounded with the lower and the apex of the left pulmonary artery. The LAD coronary artery was delineated in a 5-mm diameter. The ipsilateral and contralateral lungs were contoured out of the major airways. The contralateral breast, including all the mammary glands, was also contoured.

### Treatment Planning

We designed the VMAT plans using the Pinnacle 9.10 system, and treatment was performed on a Trilogy linear accelerator (Varian Medical Systems, Palo Alto, CA, United States) with flattening-filter–free (FFF) beams using 6-MV photons. The VMAT plan had three to four arcs with an angle ranging from 310°–320° to 135°–150°. We created two VMAT treatment plans for each patient: an FB plan and a DIBH plan. We prescribed 50 Gy in 25 fractions to the PTV FFF 6-MV photons based on the recommendations of the ICRU 83 report (29). The planning gross target volume of the tumor bed with a simultaneous integrated boost was planned to the tumor bed with 60 Gy in 25 fractions.

### Treatment Workflow

Complete treatment plan, including the plan isocenter and treatment fields, was exported in DICOM format from the TPS to the Catalyst^TM^ system. The OSMS has two modes: setup mode and treatment mode. The patients watch and control the breathing curve by EPSON glasses. Each breath hold should be within the range of the window displayed on the smart glasses (Figure 1B). As shown in Figure 1B, we defined a gating window in deep inspiration.

**Setup Mode:** All the patients breathed calmly and were initially positioned for FB using the line on the body surface made during the CT simulation. In addition, the rest of the process was performed in a breath-holding state. Before the first treatment, the OSMS was used for auxiliary positioning according to the DIBH reference image transmitted by the TPS. In addition, the offsets in the lateral (LAT), longitudinal (LONG), and vertical (VERT) directions were less than 3 mm. Cone beam CT (CBCT) was performed in position-verify using the chest wall as the alignment landmark. If the offset was more than 3 mm, the position was adjusted manually until it was considered valid for treatment. Figure 1A provide the calculation of the setup errors by the OSMS registration. The CBCT images acquired by the fast scanning technology were used to match the presented image with the DIBH reference image (CT_ref_). The result was used to adjust the baseline and breathing amplitude range consistent with the CT simulation. Then, the OSMS rescanned the patient’s body contours, making a new reference image, which we called OSMS_ref_, indicating that we entered the Catalyst^TM^ treatment mode.

**Treatment Mode:** Once the patient was positioned correctly, the Catalyst^TM^ treatment mode was entered. In addition, the OSMS was used to monitor the patient’s breathing amplitude in real time. When the patient held her breath to ensure the breathing amplitude was within the window, the first X-ray arc was released as indicated by the treatment plan. The above operation was repeated when the second X-ray arc arrived. Generally, breath holding for no less than 30 s at a time was a requirement for all the enrolled patients. Then, OSMS_ref_ was used as the reference image for each subsequent treatment, and the positioning offset was recorded and compared with the CBCT calibration offset.

### Intrafractional Displacements

In this study, we investigated intrafractional OSMS isocenter reproducibility during the RT procedure. Data about the displacement about the optical surface images were recorded in three directions, respectively (LAT, LONG, and VERT). The CBCT data used to validate the movement were also recorded. Displacements from the OSMS and CBCT registrations of the 48 enrolled cases were compared to analyze and present the reproducibility of the OSMS.

### Dosimetric Assessment

**FIGURE 2:**
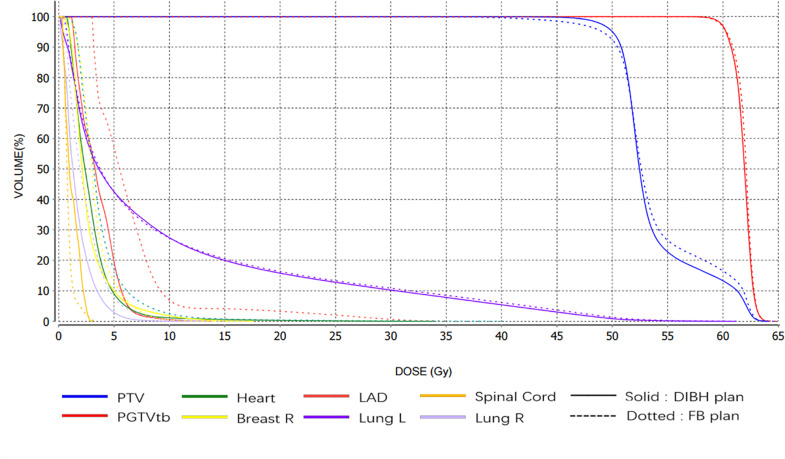
The DVH of target volume and OAR for VMAT plan with OSMS monitor in the FB and DIBH position for the same case.

Dose distribution and radiobiological endpoints were utilized to assess the dose to the target and normal tissues. Dose information of the OARs (including the heart, LAD coronary artery, lungs, and the right breast) was extracted from the dose–volume histograms (DVHs) and were compared between the DIBH-VMAT and FB-VMAT plans. Furthermore, we compared the Homogeneity Index (HI) within PTV between both plans, which was calculated as (*D*_2__%_ - *D*_98__%_)/*D*_50__%_ where *D*_2__%_, *D*_50__%_, and *D*_98__%_ are the doses received by 2, 50, and 98% of the volume, respectively (29). Figure 2 shows the DVH of the target volumes and OARs for the VMAT plan with the OSMS monitor in the FB and DIBH procedures for the same patient.

### Data Recording and Statistical Analysis

All the data in the current article were extracted from TPS software and recorded in Excel. All the data were analyzed by SPSS Statistics Software (version 25.0; IBM) using the two-sample paired *t* test. We considered a significant difference when *p* < 0.05 for all tests.

## Results

For all the enrolled cases, the median age was 54 years (range = 40–68 years). As measured with the OSMS, the mean amplitude of movement during the CT scan was 4 mm with a range of 2.5–10 mm during FB and 15 mm with a range of 12–20 mm during DIBH.

### Treatment Time

The FFF mode was utilized in clinical practical application for each DIBH case, whereas FF-mode plans were generated for each same patient. Treatment times for the two types of plan were compared. The use of FFF VMAT mode (24.6 ± 5.3 s) instead of flat beam mode (32.5 ± 15.2 s) was associated with a significant reduction (24.3% decrease) in the mean arc time (*p* < 0.05).

### Assessment of Intrafractional OSMS Isocenter Reproducibility

**FIGURE 3:**
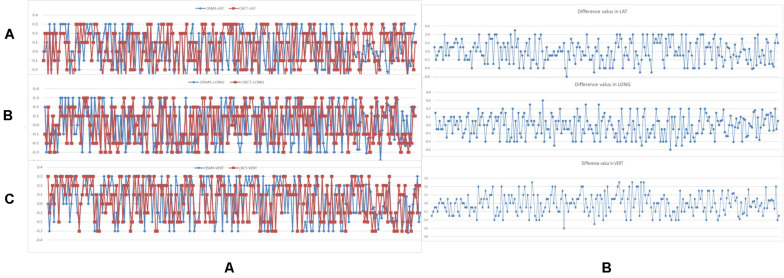
**(A)** Interfractional displacement (cm) of 240 setups in 48 breast cancer patients in the LAT, LONG, VERT direction using the OSMS and CBCT scan. The blue line shows the displacement of the OSMS scan; the red line indicates the displacement of the CBCT scan. **(B)** Difference values between OSMS and CBCT = Values(OSMS) minus Values(CBCT).

**FIGURE 4:**
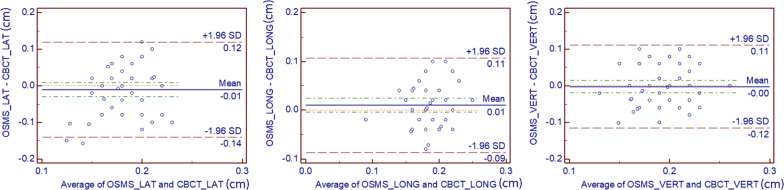
Analysis of Bland-Altman consistency in the three directions. The graphs show the mean value of 5 setups for each of the 48 patients (totally 240 setups are included).

For the 48 patients, the absolute values of the intrafractional displacements on three directions for the OSMS versus CBCT were 1.278 ± 1.135 versus 1.118 ± 0.600 mm in LAT, 1.692 ± 0.843 versus 1.106 ± 0.861 mm in LONG, and 1.418 ± 0.888 versus 0.941 ± 0.827 mm in VERT, respectively, without any significant difference (all *p* > 0.05). Figure 3 shows the intrafractional displacement in the three directions. In addition, we did the Bland–Altman consistency analysis, as shown in Figure 4.

### Volumes

Table 1 shows the mean volume (±standard deviation) of all the delineated OARs’ volumes with the FB and DIBH plans. With the DIBH plan, the heart volumes receiving irradiation were significantly smaller than those with the FB plan, whereas the volumes of the left and right lungs receiving irradiation were increased. All the mean lung volumes and left and right lung volumes increased by more than half during DIBH (*p* < 0.01), whereas the mean volumes of the heart decreased by 10.9% during DIBH (*p* < 0.05). We did not find any significant difference in the other delineated volumes in the current study.

**TABLE 1:** Mean volume (±SD) for all delineated volumes in FB and DIBH.

**Volumes**	**DIBH**	**FB**	***p* value**
PTV	872.3 ± 389.8	867.2 ± 385.5	>0.05
Heart	563.8 ± 51.1	633.1 ± 105.0	<0.05
LAD coronary artery	2.7 ± 0.8	2.8 ± 0.8	>0.05
Ipsilateral lung	1885.7 ± 454.0	1207.0 ± 391.3	<0.01
Contralateral lung	2160.7 ± 465.4	1417.4 ± 397.0	<0.01
Total lung	4035.2 ± 914.0	2589.8 ± 713.0	<0.01
Contralateral breast	653.3 ± 305.0	640.2 ± 293.0	>0.05

### Planning

**FIGURE 5:**
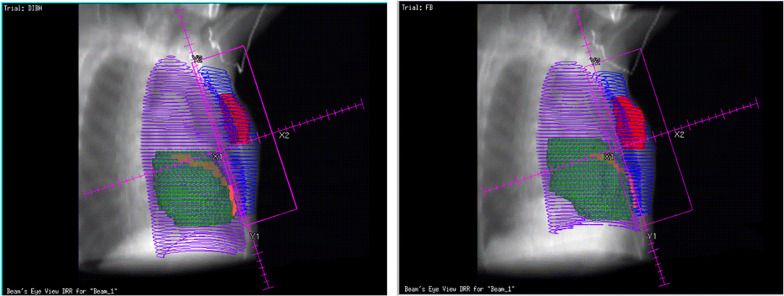
Bean’s eye views of the medial tangential field during DIBH (left) and FB (right) for a typical patient. During inspiration the lung volume (purple line) is increased, the breast (blue line) is moved cranioventrally and the heart (green) caudally In the shown case, the heart and LAD coronary artery (orange) were not included in the beam portal during DIBH.

The DVH parameters are all listed in Table 2 and shown in Figure 2, which illustrate a significantly reduced dose to the heart and LAD coronary artery in the DIBH plan. For demonstration purposes, with the same patient, we show the dose distribution and a beam’s view from the medial tangential field for the DIBH and FB plans in Figure 5. Furthermore, no significant differences were found in homogeneity, with a mean HI of 0.278 ± 0.038 for the DIBH plan compared with 0.293 ± 0.052 for the FB plan.

**TABLE 2:** Summary of treatment planning data for organs at risk, in the FB plan and DIBH plan, respectively.

**Dose**	**DIBH**	**FB**	***p* value**
**Heart**			
Mean (Gy)	3.6 ± 0.9	5.4 ± 1.6	<0.01
D2% (Gy)	13.4 ± 4.7	19.3 ± 6.1	<0.01
V5 (%)	15.9 ± 9.0	24.1 ± 8.6	<0.01
V10 (%)	4.3 ± 2.7	8.7 ± 5.0	<0.01
**LAD coronary artery**			
Mean (Gy)	3.9 ± 1.1	6.9 ± 1.8	<0.01
V10 (%)	6.4 ± 5.9	15.2 ± 9.7	<0.01
D2% (Gy)	9.0 ± 1.9	19.5 ± 5.8	<0.01
**Left lung**			
Mean (Gy)	9.5 ± 1.3	11.3 ± 1.3	<0.01
D2% (Gy)	44.9 ± 3.4	45.5 ± 3.2	>0.05
V5 (%)	42.5 ± 6.0	49.3 ± 5.0	<0.01
V10 (%)	27.1 ± 4.4	32.0 ± 4.3	<0.01
V20 (%)	16.5 ± 2.6	19.5 ± 3.0	<0.01
**Right lung**			
Mean (Gy)	1.6 ± 0.6	2.1 ± 1.1	<0.01
V5 (%)	3.9 ± 3.0	7.3 ± 4.2	<0.01
**Right breast**			
Mean (Gy)	2.1 ± 0.7	2.6 ± 1.0	<0.01
V5 (%)	5.8 ± 4.4	11.1 ± 5.0	<0.01

### Cardiac Doses

**FIGURE 6:**
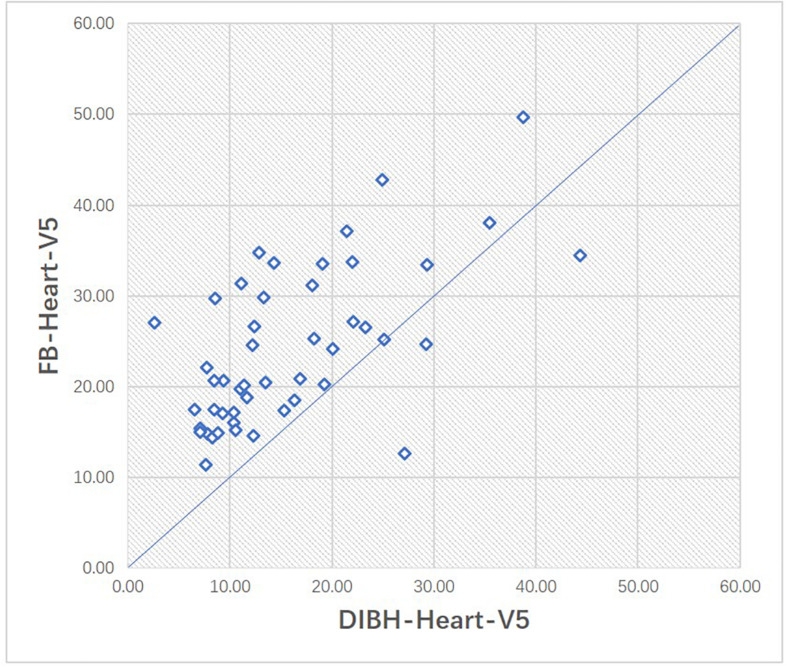
Scatter plot of the volumes of heart receiving 5Gy (V_5_) in FB and DIBH plans for each patient.

With the DIBH technique, the average mean heart dose decreased from 5.4 Gy to 3.6 Gy, showing a significant difference (*p* < 0.01). The average D2% to the heart decreased from 19.3 Gy in the FB plan to 13.4 Gy in the DIBH plan (*p* < 0.01). The mean V_5_ was also significantly reduced in the DIBH plan relative to the FB plan (15.9% vs. 24.1%, respectively, *p* < 0.01). As shown in Figure 6, we illustrate the scatter plots of V_5_ in the FB and DIBH plans for each patient. V_5_ larger than 20% was found in 30 cases (62.5%) with the FB technique and in 13 cases (27.1%) with the DIBH technique (Figure 6). The largest V_5_ to the heart in all patients was 49.65% with the FB plan and 44.36% with the DIBH plan.

We found a significant (*p* < 0.01) difference in the mean dose of the LAD coronary artery decreased from 6.9 Gy (3.11–12.9 Gy) in FB to 3.9 Gy (2.06–6.29 Gy) in DIBH plan. The average D2% to the LAD was reduced from 19.5 Gy in the FB plan to 9.0 Gy in the DIBH plan (*p* < 0.01). Moreover, the mean V_10_ to the LAD coronary artery was also significantly reduced, from 15.2% with the FB plan to 6.4% with the DIBH plan (*p* < 0.01).

### Pulmonary Doses

**FIGURE 7:**
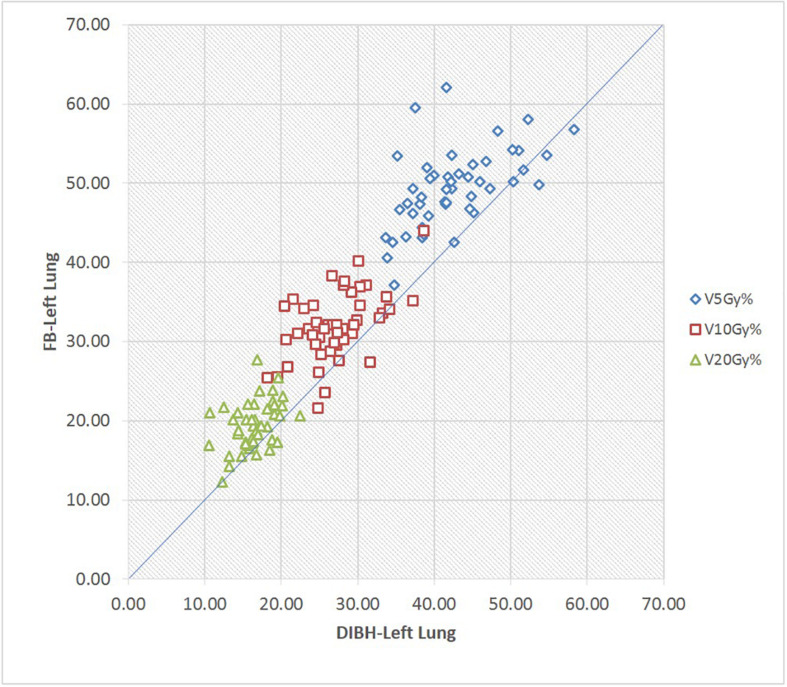
Scatter plot of the volumes of left lung receiving 5Gy, lOGy, 20Gy in FB and DIBH plans for each patient respectively.

In this study, we found a 1.8-Gy lower mean dose to the left lung with DIBH than with FB (9.5 vs. 11.3 Gy), showing a significant difference (*p* < 0.01). Our data also showed significant differences in the average V_5_, V_10_, and V_20_ to the ipsilateral lung (*p* < 0.01) (Table 2). Furthermore, the V_5_, V_10_, and V_20_ to the ipsilateral lung for each patient receiving the FB or DIBH technique are plotted in Figure 7. The largest V_5_ values with the FB and DIBH plans were 62.08 and 58.32%, respectively. A V_5_ larger than 30% was found in 25 patients (52.1%) with the FB technique, while only 11 patients (22.9%) using the DIBH technique. The V_20_ to the left lung was lower than 23% in all patients who received the DIBH plan and larger than 23% in five patients who received the FB plan. In the current study, we did not find any significant difference in D2% between the DIBH and FB plans. For the right lung, a significantly lower mean dose was found with DIBH technique than the FB technique (1.6 ± 0.6 vs. 2.1 ± 1.1 Gy, respectively, *p* < 0.01). Moreover, the average V_5_ was also reduced significantly from 3.9% with the FB plan to 7.3% with the DIBH plan (*p* < 0.01).

### Contralateral Breast

We found a 0.5-Gy lower mean dose to the contralateral breast with DIBH than with FB, showing a significant difference (2.1 vs. 2.6 Gy, respectively, *p* < 0.01), and the V_5_ was nearly half (5. 8% with the DIBH plan vs. 11.1% with the FB plan, *p* < 0.01).

## Discussion

During RT, all patients did well following audiovisual guidance and completed an excellent DIBH treatment procedure. The repeatability of the DIBH range within a 3-mm gating window was acceptable and administered well under audiovisual guidance. As reported by current studies, DIBH presented a lower OAR dose than FB in left-sided breast cancer, especially in minimizing cardiac complications (30, 31). As we know, the current study is among the first to investigate the DIBH-VMAT technique using the OSMS for breast irradiation.

Regarding the OAR, the heart, all dosimetric parameters (including the mean dose, D2%, V_5_, and V_10_) showed a better performance with DIBH-VMAT than with FB-VMAT, similar to a previous study (32, 33). As early as 2005, Korreman et al. (34) published a result about nine left-sided breast cancer patients with RT, showing that DIBH substantially reduced the doses to the heart and lung. In 2008, Stranzl and Zurl (35) also reported a significantly reduced dose to the heart with the DIBH technique in 22 left-sided breast cancer cases. Since then, additional studies on DIBH and FB have reported similar results (14, 36–38). In our opinion, the reduced heart and LAD coronary artery volume may be attributed to DIBH when increased intrathoracic pressure induced by the movement of the diaphragm and inflation of the lungs facilitates the heart far from the chest wall. Furthermore, the significantly decreased mean and V_5_, V_10_, and V_20_ of the left lung were noted with DIBH compared with FB, similar to published studies (27, 32).

In addition, in the current study, the mean and V_5_ to the right breast and right lung were nearly half with DIBH-VMAT compared with FB-VMAT. This is very different from the study published by Vikström et al. (32), in which DIBH resulted in a small dose increase to the contralateral breast. However, there was no appreciable dose difference between the DIBH-VMAT and FB-VMAT plans (*p* = 0.8849) for the right breast in the report of Swamy et al. (39). Nevertheless, our results are consistent with those from previously published studies (27, 40). Dumane et al. (41) also reported that the combination of VMAT and DIBH showed significantly lower doses to the heart, lungs, and contralateral breast. This may be attributed to the different RT technologies used. 3D conformal RT (3DCRT) with opposed beams using a flash has been the traditional technique for breast cancer. The flash aims to ensure good breast coverage, even with intrafractional or interfractional movement or in cases of breast swelling, shrinking, or deformation during treatment procedure. VMAT has the advantage of optimizing treatment plans, fast planning and RT delivery, more doses covering target tissues, and less doses to OARs (42). In addition, as reported in a published study (39), VMAT significantly improved the conformity index and HI compared with 3DCRT for the PTV of left breast RT. Moreover, the DIBH plans in the current study were generated using VMAT with 6-MV FFF beams, which allows a shorter treatment time. FFF beams have higher dose rates than flattened beams of equivalent energy, which can lead to an increased efficiency of treatment delivery, especially in conjunction with increased FFF beam energy and arc-based delivery configurations (43). In contrast, Vikström et al. (32) utilized tangential beams in their study. Under DIBH, some tissues (e.g., heart) that have been within the beam portals in FB are replaced by low-density tissues (e.g., lung). The increased dose to the contralateral breast may be attributed to the relative increase in dose to the medium and dorsolateral part of the irradiated volume, which results from increased photon beam transmission through lung tissue. VMAT is a dynamic rotational evolution of IMRT in which optimized 3D dose distribution can be emissioned in a single gantry rotation and ultimately shorten the treatment time while potentially increasing normal tissue dose sparing. VMAT produces a highly conformal dose distribution with steep dose gradients by simultaneously changing the multileave collimator position, dose rate, and gantry speed during patient treatment (44), in which case irradiation accuracy is highly dependent on the effects of positioning errors and respiratory movement, where the DIBH technique used in the present study can be beneficial. This problem does not exist in standard tangent field alone breast irradiation because the conventional tangent field utilizes wild field irradiation. However, the VMAT plan used in the current study can achieve maximum target dose coverage and minimum normal tissue exposure, which may have caused the different dose-sparing results for the contralateral breast between our study and the report of Vikström et al.

In summary, there is a significant dose reduction to OARs, including the heart, lungs, and right breast, with DIBH-VMAT. The analysis of the OAR parameters demonstrates that DIBH can provide more protection for the OAR. Similar to previous results (40), the current study indicated that DIBH can provide a stronger advantage for OAR protection when combined with VMAT. In the meantime, the OSMS can provide higher accuracy and better reproducibility (45, 46). For a patient treated with the DIBH technique, there will be several DIBH courses in an integrated treatment. Hence, this technique calls for high reproducibility for every breath hold. However, with the prolonged treatment time, the reproducibility of each breath hold will decrease from the previous DIBH treatment. Increased instability will lead to increased unexpected radiation exposure to OARs, which need protection during RT (27). A good treatment technique could provide a shorter total fractionated RT time (setup time and treatment time in the current study), which can assist the patient in accommodating the whole treatment procedure well in a shorter time and improve treatment quality. We previously reported (17) that the OSMS is a simple, fast, reproducible, and accurate solution for patient setup and can minimize random day-to-day setup errors. The data presented in the current study show that it took approximately 2 min to complete the position-verify, whereas less than 20 s with OSMS. Shorter time assists in reducing the movement caused by poor tolerance in the treatment delivery. Meanwhile, OSMS can simplify the position-verify process and provide a real-time position and breathing information during the treatment without prolonging the total fractionated RT time. “Real-time monitoring” is the greatest strength of the OSMS; when the patient’s breathing amplitude exceeds the gating window, the radiation beam is closed to prevent mismatch. Thus, DIBH-VMAT with the OSMS can provide not only the advantage of OAR (including the heart, lung, and other important tissues) sparing but also a shorter position-verify time to assist the patient proceed through treatment effortlessly. Furthermore, we will perform long-term follow-up to confirm the clinical advantages of the presented favorable dosimetric benefit, as well as OSMS in RT for left-sided breast cancer cases.

## Conclusion

DIBH-VMAT with OSMS is very feasible in daily practice with excellent patient compliance in our single-center experience. Note that OSMS is an effective tool that may allow easier-to-achieve precise positioning and better and shorter position-verify time. Meanwhile, compared with FB, DIBH was characterized by lower doses to OARs, which may reduce the probability of cardiac and pulmonary complications in the future.

## Data Availability Statement

All datasets presented in this study are included in the article/supplementary material.

## Ethics Statement

The studies involving human participants were reviewed and approved by Institutional Review Boards of Yantai Yuhuangding Hospital and Academy of our Medical Sciences. The patients/participants provided their written informed consent to participate in this study.

## Author Contributions

WZ, RL, and DY analyzed the data and wrote the manuscript. YS and ZM helped with the statistical analysis and editing the manuscript. WD critically revised the manuscript. All authors contributed to the article and approved the submitted version.

## Conflict of Interest

The authors declare that the research was conducted in the absence of any commercial or financial relationships that could be construed as a potential conflict of interest.

## Abbreviations

BC: breast cancer
CTV: clinical target volume
CVD: cardiovascular disease
DIBH: deep inspiration breath hold
DVH: dose–volume histograms
ECOG-PS: Eastern Cooperative Oncology Group performance status
FB: free breathing
IMRT: intensity-modulated radiation therapy
LAD: left anterior descending
OAR: organs at risk
OSMS: Optical Surface Management System
PTV: planning target volume
VMAT: volumetric modulated arc therapy.
